# Addressing Cancer Screening Disparities in Little Haiti, Miami, USA: A Literature Review on Barriers and Solutions

**DOI:** 10.7759/cureus.84651

**Published:** 2025-05-22

**Authors:** Hope M Cherian, Craig Warlen, Alan Martin, Daniel Ajabshir, Nana Aisha Garba

**Affiliations:** 1 Humanities, Health, and Society, Florida International University Herbert Wertheim College of Medicine, Miami, USA

**Keywords:** breast cancer, cancer screening, cervical cancer, colorectal cancer, demographic factors, little haiti, screening adherence, screening guidelines, socioeconomic factors, west little river

## Abstract

Breast, cervical, and colorectal cancers pose significant health risks to women, with early screenings proven to reduce mortality. However, disparities in screening adherence persist, particularly in underserved communities like Little Haiti in Miami. This review examines screening rates in Little Haiti, identifying barriers to adherence and evidence-based interventions to improve participation. Screenings significantly reduce cancer mortality. Despite these benefits, screening rates in Little Haiti remain below national averages. Socioeconomic and cultural factors, including language barriers, misconceptions, and limited health literacy, further hinder adherence. Effective strategies identified in this review to address these disparities include reminder systems, self-administered human papillomavirus (HPV) tests, and targeted outreach. Programs like Florida International University’s Neighborhood Health Education Learning Program (NHELP) initiative improve access by integrating preventive care with education. However, research gaps remain in evaluating culturally specific interventions. Future studies should explore native-language education, mobile screening units, and technology-driven solutions to enhance adherence. This review highlights the need for targeted strategies to ensure equitable access to life-saving cancer screenings in underserved communities.

## Introduction and background

Background and significance

Breast and cervical cancers are among the most frequently diagnosed chronic diseases in women. According to the American Cancer Society, in 2023, it is estimated that 297,790 new cases of breast cancer were reported in women, as well as 13,960 new cases of invasive cervical cancer, totaling well over 300,000 new diagnoses in the United States [1]. Within the state of Florida, the numbers of new cervical and breast cancer diagnoses were estimated to be 23,160 and 1,170, respectively [2]. Reported national incidence rates for colon cancer were 106,970 new cases, while the new cases for rectal cancer were 46,050 nationally and 11,920 for the state of Florida [2]. According to a randomized controlled trial (RCT) conducted in the United Kingdom by Duffy SW et al., a significant reduction in breast cancer mortality was found between women who had begun mammographic screenings at 40 years of age as opposed to the standard of care, which was previously 50 years [3]. A similar association between a reduction in mortality and increased screening can be seen with cervical cancer, according to a systematic review by E.E. Jansen et al. of European studies, which showed a mortality reduction for women attending organized screenings for cervical cancer vs non-attenders between 41% and 92% across seven studies [4]. A reduction in colorectal cancer mortality has also been associated with effective colorectal screenings, as shown by a systematic review and meta-analysis conducted by Zheng et al., which showed a 10-year colonoscopy reduced mortality by 73% [5]. These studies and reviews suggest that early detection of breast, cervical cancer, and colorectal cancer can reduce long-term mortality and, therefore, emphasize the importance of identifying any healthcare disparities that may exist in screening for these cancers.

Ensuring access to healthcare and a consistent source of screening guidance is crucial for meeting recommended cancer screenings. Therefore, it is imperative to assess health disparities in communities with limited healthcare access or availability of screenings. This review focuses particularly on those living in Little Haiti and its geographical proxy, West Little River. This community was selected as it corresponds to one of the underserved areas actively serviced by Florida International University Herbert Wertheim College of Medicine’s Neighborhood Health Education Learning Program (NHELP). NHELP was founded in 2010 as part of an initiative to blend medical education with primary and preventive care services offered by the school's multidisciplinary healthcare team, including physicians, nurses, physician assistants, social workers, and students from all disciplines in the care of a household. Among providing services such as offering health education, facilitating applications for legal/social aid, and following up on health concerns between provider visits, medical students have a unique opportunity to closely engage with a household and emphasize the importance of cancer screenings. This review may not only serve as an investigation into the cancer screening practices of Little Haiti, but also as a knowledge base for students and providers to use as a tool in emphasizing adherence to life-saving screening methods. As Little Haiti corresponds to a neighborhood as opposed to a city, there is no specific data corresponding to the area. Using West Little River as a proxy reveals a reported rate of cervical and breast cancer screenings of 77.3% and 76.5%. This is lower than the Miami-Dade County average (80.1%) and the US national average (82.8%) for cervical cancer, while the breast cancer screening rate is higher than the Miami-Dade average (72.6%) but lower than the US national average (78.2%) [6]. When looking at colorectal cancer, the reported screening rate in West Little River in adults between 50 and 75 years old, which corresponds to the United States Preventive Services Task Force (USPSTF) recommended guidelines, is only 66.8%, which is lower than the US national average (72.4%) and the Miami-Dade County average (69.0%) [6]. Across the survey measures used to screen for cancer incidence rate, there is less adherence to recommended guidelines in Little Haiti as compared to the average in the United States and Miami-Dade County. Further investigation is needed to evaluate this gap in adherence and discuss the disparities and potential resolutions to remedy the differences that currently exist in these communities.

Across the literature, there have been several studies completed to investigate potential disparities that may exist across socio-economic, ethnic, and cultural lines. One such study was a meta-analysis that investigated ethnic minority status and receiving a mammogram within the past 2 years among American women over 50 years old [7]. The study found that African Americans were screened less than Non-Hispanic Whites marginally (OR 0.87, 95% CI: 0.75-1.00). There were larger discrepancies when comparing Hispanics (OR 0.63, 95% CI: 0.39-0.99) and Asian/Pacific Islanders (OR 0.63, 95% CI: 0.39-0.99) to Non-Hispanic Whites. When controlling for socio-economic factors, the differences were no longer significant. While the influence of insurance status was discussed, there is no comment on how participants funded the mammograms. This study strongly suggests that socio-economic factors play a significant role in influencing whether a person receives adequate screening, and that there may be a link between socioeconomic status and screening adherence.

Another systematic review and meta-analysis investigated the association between immigrant or citizen status in women and cervical cancer screening completion. It was found that immigrant women had a significantly lower participation rate than citizen women [8]. Although this study suggests that significant health disparities exist predicated upon migrant status, this systematic review consisted mainly of studies conducted in Europe, which weakens its external applicability to populations in the United States. In an observational study conducted in households in Little Haiti, it was found that Non-Hispanic Black Households had 63% higher odds of adherence to breast cancer screening guidelines compared with Haitian households, although this was only a borderline significant disparity (OR=1.63, P=0.11) [9]. This study, conducted by Wilcox et al., sought to investigate subgroups within the Black community, such as Haitians, to explore potential disparities that can then be investigated further through individualized screening studies to identify the reasons behind the lower screening adherence rates seen in Little Haiti.

A separate secondary data analysis study assessing the association between source of healthcare and adherence to cancer screening in Little Haiti found that households with gaps in insurance coverage were significantly less likely to adhere to scheduled mammograms (OR=0.40, 95% CI: 0.17-0.97) or pap smears (OR=0.28% CI: 0.13-0.58) [10]. This study suggests that inadequate healthcare coverage can be a determining factor in whether one adheres to screening guidelines or not. In the same study, it was found that when adjusting for the head of household’s education and insurance gap, households without a regular source of care were approximately 70% less likely to adhere to colorectal cancer screenings (96% CI: 0.15-0.80) [10]. Having access to health insurance and a regular source of receiving cancer screenings is suggested to have a considerable impact on whether a household adheres to the current screening guidelines. It is also important to consider other factors that contribute to the current disparities, such as cultural influences regarding screening procedures, knowledge of the appropriate screenings, and socioeconomic factors. In a study conducted with the goal of assessing screening adherence among women in two distant immigrant communities residing in Miami-Dade County, either in Little Haiti (Haitian) or Hialeah (Cuban), it was found that adherence to cervical cancer screenings was low in both [11]. Furthermore, the study found a significant correlation between having health insurance and more recent mammograms. Additional disparities were observed between the Hispanic and Haitian participants. Women in Little Haiti exhibited a higher likelihood of harboring misconceptions about cervical cancer, adopting a more fatalistic perspective toward their diagnosis, and were significantly less informed about human papillomavirus (HPV) compared to their counterparts in Hialeah [11]. There are several possible explanations for the difference in education about the health screenings among the two different populations, which can then be plausibly extrapolated to other distinct immigrant communities. One such possibility is the lack of educational resources targeted towards Haitian woman in their native language or a lack of adequate community engagement to properly educate the community about screening guidelines, as well as a general lack of prevention-focused healthcare resources in the area. It is important to further assess the efficacy of current community programs and guidelines to address these disparities and develop new, innovative solutions to address these concerns.

Cultural and social contexts

As of 2022, the breakdown of West Little River racial and ethnic demographics (compared to the United States) are as follows: Non-Hispanic Black or African American 44.7% (12.2%); White Hispanic 23.1% (8.73%); Multiracial Hispanic 12.6% (3.82%); Other Hispanic 11.1% (5.21%); Hispanic Black 4.4% (0.36%); Non-Hispanic White 3.27% (59.4%) [12]. The percentage of foreign-born residents of West Little River is 44% compared to 13.6% nationally. The poverty rate of West Little River is 22.4%, with a national poverty rate of 12.6% [12,13]. Twenty-three-point one five percent of the West Little River population did not finish high school, while 8.9% of US residents did not finish high school [12, 13]. The uninsured rate of West Little River is 22.3% compared to a national noninsurance rate of 8.77% [12,13]. 51.1% of individuals reside in Spanish-speaking households and 8.97% in Haitian Creole-speaking households compared to 13.3% and 0.28%, respectively, nationally [14].

In the West Little River community, with predominantly Black and Hispanic populations and lower education levels than the national average, adequate access to health education and screening services is imperative to promote a healthy community. Specific community barriers to both access to screening and follow-up care include lack of internet access, health insurance, and citizenship/immigration status. In West Little River, 19% of households are without a broadband internet subscription. Patients without access to the internet are more likely to have low health literacy; therefore, less likely to pursue preventative care and screenings [15]. High rates of uninsured people in these communities also lead to less adherence to preventive healthcare and screenings. In West Little River, 22.4% of individuals under age 65 are uninsured. A crucial factor affecting these rates of health insurance is the citizenship status of many individuals in the community. In West Little River, 18.9% of individuals are noncitizens compared to the national average rate of 6.6%. Disparities in health insurance coverage and healthcare access for noncitizen immigrants are well-documented [16]. Furthermore, numerous studies have been conducted identifying predictive factors regarding utilization and follow-up to preventative health and screening programs. A colorectal cancer screening study found results showing lower follow-up rates in African American patients, patients of Hispanic ethnicity, and patients with a low level of education [17]. Addressing these disparities requires targeted interventions to improve accessibility, expand health insurance coverage, and provide culturally and linguistically appropriate health education and services. By tackling these barriers, it is possible to enhance health outcomes and ensure more equitable access to essential healthcare services in the West Little River community.

Table 1 presents a summary of the factors influencing cancer screening rates in West Little River.

**Table 1:** Summary of Factors Influencing Cancer Screening Rates in West Little River (Proxy for Little Haiti)

Category	Key Factors
Demographic Disparities	Screening rates lower than U.S. and Miami-Dade averages. Lower follow-up among African American and Hispanic populations.
Socioeconomic Status	High poverty rate (22.4%) compared to national average (12.6%). Lower education levels (23.15% did not finish high school).
Immigration/Citizenship	44% foreign-born; 18.9% noncitizens (vs. 13.6% and 6.6% nationally). Noncitizen status associated with lower access to screenings and insurance.
Language & Literacy Barriers	8.97% speak Haitian Creole; 51.1% speak Spanish at home. Limited availability of educational resources in native languages.
Cultural Beliefs	Cultural attitudes and mistrust may affect willingness to engage with screening services.
Healthcare Access	High uninsured rate (22.3%) vs. national average (8.77%). Gaps in insurance linked to lower screening adherence.
Health Literacy & Education	Misconceptions about cervical cancer, fatalistic views, and low human papillomavirus (HPV) awareness more prevalent among Haitian women.
Technology Access	19% of households lack broadband internet linked to lower health literacy and reduced access to screening info.
Regular Source of Care	Lack of regular healthcare source associated with ~70% lower adherence to colorectal cancer screening.

Study objectives

This scoping review seeks to determine the prevalence of cancer screening in West Little River (Little Haiti) and identify potential disparities to provide a framework for the development of additional community resources to improve cancer screening compliance. Here, the current data regarding the adherence prevalence towards breast, cervical, and colorectal cancer will be examined and compared to greater Miami-Dade County and the United States. Data evaluating the efficacy of these cancer screenings in reducing the mortality rate will also be discussed. Finally, interviewing frameworks, diagnostic modalities, and community interventions will be explored that have the potential to enhance patient adherence in Little Haiti. Beyond this scoping review, the goal of this review will be to examine gaps in the current research and examine potential avenues for increasing cancer screening adherence in Little Haiti and beyond.

## Review

Search strategy

This literature review was conducted on PubMed and focused on articles discussing causative factors for decreased adherence to cancer screening recommendations, as well as solutions that were shown to be effective at increasing adherence with regular cancer screening.

As many studies have been conducted in this area, inclusion criteria were set to include only systematic reviews and meta-analyses of research to obtain aggregated evidence for each search conducted. As the issue of adherence to cancer screening recommendations is not a new phenomenon, it was decided not to set a limit on when these papers were published. Papers were excluded if they were too specific to the population they targeted. For instance, a paper that focused exclusively on healthcare providers in the community was not included. Publications with this level of specificity were excluded, as the primary objective of this report is to ensure that the analyzed data can be applied to the broader community of Little Haiti.

**Figure 1:**
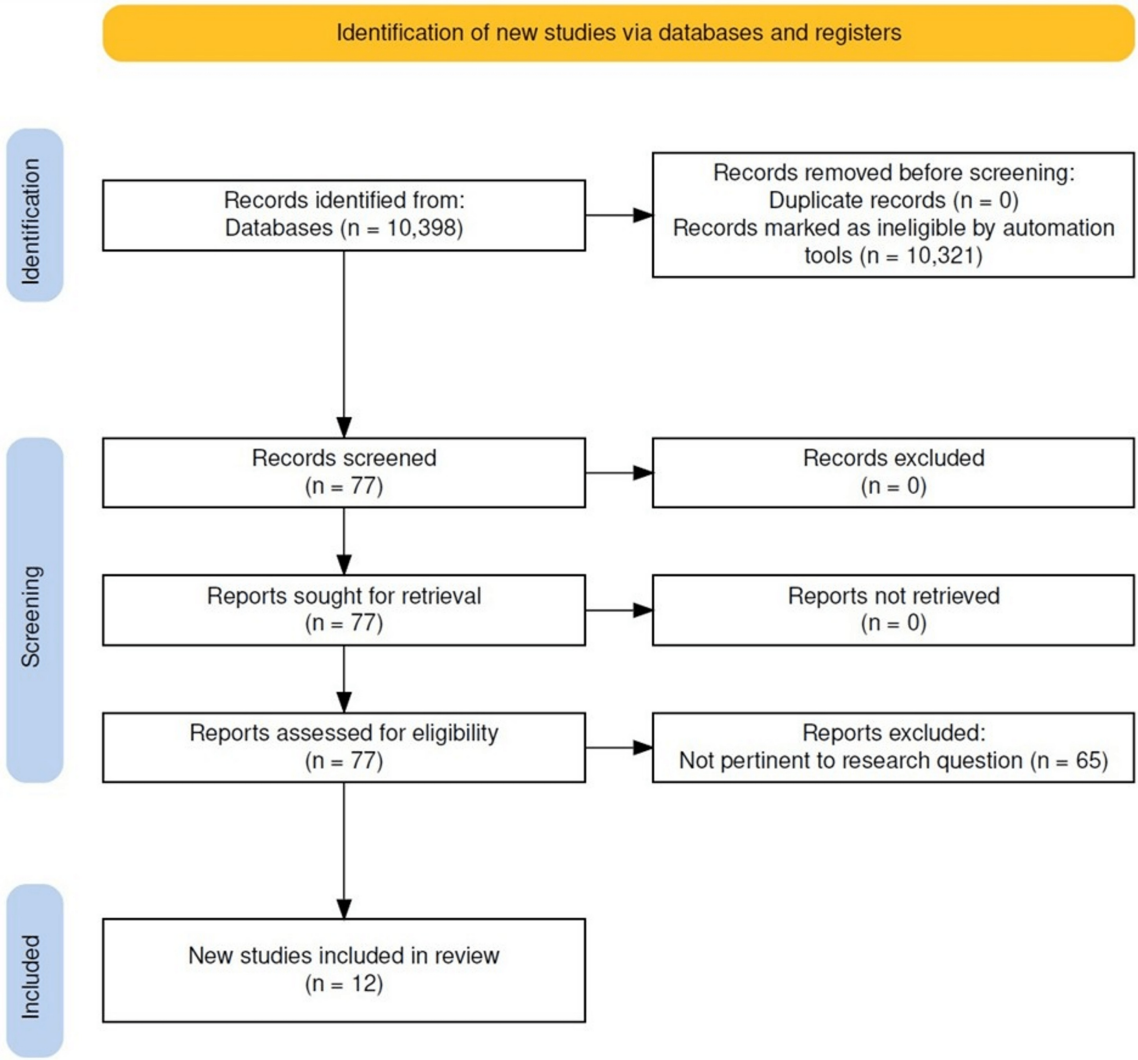
Preferred Reporting Items for Systematic Reviews and Meta-Analyses (PRISMA) flow diagram.

The MeSH search, ("Guideline Adherence"[Mesh]) AND "Early Detection of Cancer"[Mesh] OR "Patient Compliance" [Mesh]) AND ("Breast Neoplasms"[Mesh] OR "Uterine Cervical Neoplasms"[Mesh] OR "Colorectal Neoplasms"[Mesh]) was conducted first. A subsequent search was conducted using the keywords (cancer screen) AND (compliance). To identify causes and solutions for decreased adherence with breast cancer screening, the search was adjusted again to use keywords (mammogram) AND (compliance). As seen in Figure 1, there were initially 10,398 results. 10,321 were not included because they were not systematic reviews or meta-analyses. Seventy-seven articles were screened. Of these, 65 were excluded because they were not pertinent to our research question. For example, some studies had study populations that were substantially different from ours or did not investigate screening adherence as an outcome. In total, there were twelve articles included in this review.

Data charting and synthesis

Table 2 presents a data chart of the 12 research studies identified for inclusion in this review.

**Table 2:** Data chart of identified research

Title [reference no.]	Author	Publication Year	Country	Aim/Purpose	Study Population & Sample Size	Methodology/Methods	Intervention type/duration, comparator, outcome measures (if applicable)	Key findings that relate to the research question(s)
Effective interventions to facilitate the uptake of breast, cervical and colorectal cancer screening: an implementation guideline [18]	Brouwers MC, De Vito C, Bahirathan L, et al	2011	Canada	To develop a guideline to answer the question: What interventions have been shown to increase the uptake of cancer screening by individuals, specifically for breast, cervical, and colorectal cancers?	66 Randomized Controlled Trials	An expert panel first assessed studies for inclusion in the development of a potential guideline for increasing cancer screening adherence. This guideline draft was then sent to Cancer Center Ontario's Report Approval Center for a first round review, then to eight Canadian and American external reviewers for a second round of review.	This meta-analysis examines several interventions to increase cancer screening adherence, and assesses combined intervention strategies using the AGREE II framework, an internationally designed framework intended to assess the quality of proposed guidelines [19].	This study notes that a majority of the 66 studies included in the meta-analysis were of moderate to poor quality. Nevertheless, the intervention guidelines proposed based on this study were evaluated positively using the AGREE II framework. The interventions included in these guidelines were client reminders, reduction of structural barriers, and having providers give their own assessments and feedback on the results of the screenings.
Methods to increase participation in organised screening programs: a systematic review [20]	Camilloni L, Ferroni E, Cendales BJ, et al.	2013	Italy	A comprehensive systematic review of interventions was conducted to increase participation in organized cervical, breast, and colorectal screening programs, using the standard invitation letter as comparator for all the proposed interventions.	69 Randomized Controlled Trials, Experimental Studies, and Before and After Studies	Multi-database search of articles that fit within defined inclusion and exclusion criteria, followed by a quality assessment utilizing the CONSORT list, a study classification, then finally data analysis using Review Manager 5 via the Cochrane Collection.	This systematic review examined a variety of interventions that attempted to increase adherence to cervical, breast, and colorectal cancer screening guidelines in European populations.	The study identified several effective interventions to enhance screening adherence. Postal and telephone reminders proved effective, increasing the relative incidence of screening adherence by 1.33 to 1.71, depending on the specific cancer screen. Invitations to undergo screening, accompanied by the signature of the patient's general practitioner, resulted in a relative incidence ranging from 1.13 to 1.20. Additionally, scheduling an appointment for the screening, as opposed to leaving it open-ended, showed a relative incidence of screening adherence ranging from 1.26 to 1.79, depending on the specific cancer screen. Notably, self-administered tests for cervical cancer screening exhibited a high relative incidence of screening adherence at 2.37.
Self-collected HPV testing improves participation in cervical cancer screening: a systematic review and meta-analysis [21]	Racey CS, Withrow DR, Gesink D.	2013	Canada	To determine the extent to which providing self-collected human papillomavirus (HPV) testing increases screening participation in women who are never or underscreened for cervical cancer.	8 European and 2 North American Studies	"A systematic literature review conducted in the databases Medline and Embase identified articles examining the use of HPV self-testing on cervical cancer screening participation. A meta-analysis using a random-effects model was used to calculate the relative compliance, with an intent-to-treat analysis of HPV self-testing compared to Pap testing, with 95% confidence intervals (CI). All statistical tests were two-sided."	This study focused on the intervention of a self-administered human papillomavirus (HPV) test as a method of improving cervical cancer screening adherence. For each study in the review, the control groups were provided standard treatment options, and the experimental groups were provided with self-administered HPV tests.	The overall relative adherence of the self-administered HPV test compared to the pap smear was 2.14 with a 95% confidence interval spanning from 1.3 to 3.52.
Meta-analysis: adherence to colorectal cancer screening and the detection rate for advanced neoplasia, according to the type of screening test [22]	Hassan C, Giorgi Rossi P, Camilloni L, et al.	2012	Italy	"To perform a meta-analysis on adherence and detection rates of CRC screening tests."	14 studies providing data on 197,910 subjects.	"Relevant publications were identified by MEDLINE/EMBASE and other databases for the period 1999-2012. A previous systematic review was used for the period before 1966-1999. Randomized control trials (RCTs) and controlled studies including a direct comparison of the uptake rates among different options for colorectal cancer (CRC) screening were included. Adherence and detection rates for advanced neoplasia and cancer were extracted. Risk for bias was ascertained according to Consolidated Standards of Reporting Trials (CONSORT) guidelines. Forrest plots were produced based on random-effect models."	This study focuses on colorectal cancer screenings and attempts to define how well patients adhere to colorectal cancer screenings based on the screening method utilized and analyzes that against how effective each screening method is at detecting colorectal cancer.	Across 14 papers, this study finds a lower adherence rate to endoscopic screening as opposed to fecal testing, with a relative incidence of screening adherence of 0.67; however, endoscopic screening has a much higher detection rate for advanced neoplasia, with a relative incidence of detection of 3.21. Between the fecal cancer screens, Fecal Immunochemical Test (FIT) was found to be superior to guaiac-based Fecal Occult Blood Test (g-FOBT) screening with regards to screening adherence, neoplasia detection, and cancer detection, with respective relative incidences of 1.16, 2.28, and 1.96.
The effect of mammography pain on repeat participation in breast cancer screening: a systematic review [23]	Whelehan P, Evans A, Wells M, Macgillivray S.	2013	United Kingdom	"1. What is the range, nature and quality of the current evidence? 2. How commonly do women choose not to re-attend for breast screening because of a prior painful mammogram? 3. Are there any sub-groups of women who are more likely to avoid breast screening because of mammography pain?"	20 studies from Europe and North America	"Searches were run in 10 online databases: Medline, Embase, PsycINFO, CINAHL, ASSIA, Cochrane Database of Systematic Reviews, Sociological Abstracts, SSCI, SCI, and NHS Cancer Screening Programmes' online literature database." This was followed by screenings of identified papers to ensure they conformed with inclusion and exclusion criteria. Data extraction was performed independently by two of the authors, who checked each other's findings.	The study examined both the causation and association between mammography pain and re-attendance rates.	The proportion of patients who did not re-attend for mammography due to pain ranged from 11% to 46% across five selected studies. Evidence for an association between pain experienced at a previous mammogram and subsequent rates of re-attendance suggests that women who previously experienced pain are more likely than those who did not to fail to re-attend, with a recurrence risk of 1.34 (95% CI: 0.94–1.91). It was further highlighted that the quality of the communication between mammography staff and clients affected reported pain.
Motivational Interviewing to Improve the Uptake of Colorectal Cancer Screening: A Systematic Review and Meta-Analysis [24]	Long NN, Lau MPXL, Lee ARYB, Yam NE, Koh NYK, Ho CSH	2022	Singapore	"This review synthesizes the efficacy of motivational interviewing in promoting uptake of colorectal screening modalities and is the only review so far that examines motivational interviewing for colorectal cancer screening alone."	14 Randomized Controlled Trials with a low to moderate risk of bias	"A systematic review and meta-analysis was conducted to examine the effects of motivational interviewing for colorectal cancer screening. PubMed, EMBASE, CENTRAL, PsycINFO, and CINAHL were searched to identify eligible studies from inception to June 2021 and selection criteria was defined." Cochrane Risk of Bias tool was used for bias assessment. Data analysis was conducted using the Laird random effects model.	This study explored the use of motivational interviewing as an intervention to improve adherence with colorectal cancer (CRC) screenings, with control groups receiving standard recommendations for CRC screening.	Overall, the study reported a relative incidence of cancer screen adherence of 1.3 among groups who received motivational interviewing compared to groups who did not, meaning that patients who underwent motivational interviewing to discuss CRC screening were 1.3 times more likely to adhere to screening recommendations compared to those who did not.
Effect of physician reminders on preventive care: meta-analysis of randomized clinical trials [25]	Austin SM, Balas EA, Mitchell JA, Ewigman BG	1994	United States	"To assess the clinical value of the physician reminder, an information intervention, in increasing compliance for selected preventive health care measures."	10 Randomized Controlled Trials from the Columbia Registry.	A systematic review of randomized controlled trials in the Columbia registry followed by a meta-analysis utilizing the odds ratio method to compare utilization.	The intervention in this study was a reminder to patients from a physician to receive the preventive healthcare measures of cervical cancer screening and tetanus immunization. The control group was not provided a reminder.	The analysis found that physician reminders are an effective information intervention and can improve adherence to cervical cancer screening. When examining 3 randomized control trials, the overall odds ratio of cervical cancer screening adherence was significant (1.180, 95 percent Cl: 1.020 to 1.339).
Experiences of cervical screening and barriers to participation in the context of an organised programme: a systematic review and thematic synthesis [26]	Chorley AJ, Marlow LA, Forster AS, Haddrell JB, Waller J.	2017	United Kingdom	"This systematic review synthesises the qualitative literature on women's perceptions and experiences of cervical screening in the context of an organised call-recall programme, in order to understand the barriers to informed uptake."	39 papers from the UK, Australia, Sweden and Korea	"We searched nine databases for English language peer-reviewed publications reporting on qualitative data from screening-eligible women, exploring barriers to cervical screening in countries that offer a nationally organised call-recall programme. Evidence was integrated using thematic synthesis."	This paper focused on identifying the distinct reasons for why women were resistant to consistently completing cervical cancer screenings. As such, there were no control or experimental groups for this study.	Factors that were identified to increase resistance to screening adherence included questioning of relevance and value to the patient, the physical and emotional burden of undergoing the screening process, the perceived threats of the screening process, and whether patients believed they would act on the results of the screening.
Impact of provider-patient communication on cancer screening adherence: A systematic review [27]	Peterson EB, Ostroff JS, DuHamel KN, et al.	2016	United States	"To systematically review studies that focused on the role of provider-patient communication in screening behavior."	35 articles	Preferred Reporting Items for Systematic Reviews and Meta-Analyses (PRISMA) guidelines were utilized, and data collection included the following databases: PubMed, PsycINFO via OVID, Cochrane, and EMBASE.	This paper investigated the role of provider-patient communication in screening behavior for cervical, breast, and colorectal cancer. Literature included assessed provider recommendations, quality, and content of provider-patient communication about screening, and interventions to improve provider-patient communication about screening and screening behaviors. There were no control or experimental groups for this study.	A positive association was present between provider recommendation and patient screening adherence in almost all studies across varying types of populations and types of cancer screened. For breast cancer and Pap screening studies, talking about the screening, provider enthusiasm and explanations, elicitation of barriers, and responsiveness to patient concerns improved adherence. Generally, colorectal cancer screening was positively correlated with provider encouragement and shared and informed decision-making. Interventions were some form of communication skills training or screening education for providers. This was similarly generally positively associated with increased patient screening adherence.
The effectiveness of mailed patient reminders on mammography screening: a meta-analysis [28]	Wagner TH.	1998	United States	To compare the effectiveness of mailed patient reminders at increasing mammography screening.	16 published articles	"Sixteen published articles met the inclusion criteria and were included in the meta-analysis. To assess the association between reminders and mammography screening, the Mantel-Haenszel odds ratio (OR) was calculated."	The intervention employed in this study was mailed patient reminders. Patient reminders were either generic reminders or tailored to the individual patient. The control group was patients who did not receive any reminder. All studies included in this analysis were published randomized controlled trials. An important exclusion criterion to note was studies that provided non-mailed reminders to patients.	Women receiving reminders are >50% more likely to get a mammogram. Women receiving tailored letters were 85% more likely than those who received generic reminders.
Strategies for increasing participation in mail-out colorectal cancer screening programs: a systematic review and meta-analysis [29]	Goodwin BC, Ireland MJ, March S, et al.	2019	Australia	To systematically review interventions applied to increase fecal occult blood test (FOBT) kit return, specifically in population mail-out programs.	30 published studies	Preferred Reporting Items for Systematic Reviews and Meta-Analyses (PRISMA) guidelines for systematic reviews were utilized. Database searches of PubMed, PsycINFO, Scopus, CINAHL, and ProQuest Dissertations and Theses. Risk of bias was assessed using the Cochrane Risk of Bias tool.	Interventions for increasing fecal occult blood test (FOBT) kit return included digital reminder, added print materials, behavior priming, advance notice, simplified testing procedure, general practitioner endorsement, and telephone contact. The control group was a standard invitation without any additional information or endorsement.	Four key intervention strategies included advance notification, GP endorsement, telephone contact, and the simplification of testing procedures. Interventions involving telephone contact were associated with the highest average increase in the rate of kit. It was noted that automated contact through the form of a recorded message, email or text message reminder was not as successful and led to decreased participation in one instance.
A meta-analysis of computer-tailored interventions for health behavior change [30]	Krebs P, Prochaska JO, Rossi JS.	2010	United States	"This meta-analysis focuses on interventions that tailored feedback to individual users by means of computer algorithms, regardless of whether the feedback was delivered via print, telephone, or computer terminal."	88 unique studies including 106,243 participants	"The electronic databases PsycInfo, PubMed, CINAHL, and the Cochrane library were searched for studies using following terms: “(tailor*) and (compute* OR feedback OR individualized)”, “expert system”, “e-health AND (tailor* OR feedback OR individualized)”." Hedges G was used for the effect size statistic.	The intervention in this study was computer-tailored communication. Tailored feedback uses computer algorithms to send individualized feedback, regardless of whether the feedback was delivered via print, telephone, or computer terminal. The control group was a non-tailored comparison group.	Computer-tailored interventions resulted in 56% adherence versus 50% in control groups for receiving at least bi-annual mammography screening. There were no significant differences found between the communication channels of print, computer, or automated phone.

Results

Study Characteristics

The scoping review encompasses a diverse array of studies focused on barriers to participating in cancer screenings as well as interventions and methodologies aimed at increasing participation in cancer screening programs. Twelve studies were fully reviewed based on the search criteria previously described. The publication dates range from 1994 to 2023. These studies are international in scope, with research conducted in Canada, Italy, Singapore, the United Kingdom, and the United States. The studies included were systematic reviews and meta-analyses, many of which analyzed Randomized Controlled Trials (RCTs). For instance, a Canadian study led by Brouwers et al. [18] reviewed 66 RCTs to develop guidelines for cancer screening interventions. In contrast, an Italian meta-analysis by Hassan et al. [22] assessed colorectal cancer screening adherence using data from 14 different studies.

The study populations and sample sizes varied widely across the reviewed literature. Some studies, such as the meta-analysis involving 197,910 subjects, provided broad insights into adherence to colorectal cancer screening [22]. Others focused on more specific interventions, such as the self-administered HPV testing studied by Racey et al. [21], which included eight European and two North American studies. Another study by Whelehan et al. [23] analyzed the impact of mammography pain on repeat participation, using data from 20 studies conducted in Europe and North America.

The methodologies employed in these studies were rigorous and multifaceted. Systematic literature reviews and multi-database searches were common, often involving databases like MEDLINE, Embase, and Cochrane Library. Studies typically utilized comprehensive search strategies followed by expert panel assessments to ensure the relevance and quality of the articles included. For example, Camilloni et al. [20] conducted a systematic review that included 69 RCTs, experimental studies, and observational studies to identify effective interventions for increasing participation in organized cancer screening programs.

The types of interventions studied were diverse, ranging from educational strategies and organizational changes to technological innovations. Educational interventions often focused on increasing awareness and knowledge about the importance of screening, while organizational strategies aimed to streamline the screening process and make it more accessible. Technological innovations, such as the self-administered HPV testing, represented significant advancements in screening methodology. Outcome measures typically focused on screening adherence, patient satisfaction, and the overall effectiveness of the intervention strategies. For example, Racey et al. [21] found that self-collected HPV testing significantly improved participation in cervical cancer screening. Similarly, interventions aimed at managing pain, as studied by Whelehan et al. [23], were linked to increased repeat participation in mammography. They found that addressing mammography pain encouraged repeat participation, a critical factor in the success of breast cancer screening programs.

Key findings from these studies consistently highlighted the effectiveness of certain interventions in improving screening adherence. Brouwers et al. [18] noted that the majority of the 66 studies reviewed reported positive outcomes from various intervention strategies. Camilloni et al. [20] identified several effective interventions, including reminder systems and educational materials, which significantly increased participation rates. Hassan et al. [22] found a lower adherence rate to colorectal cancer screening among certain groups based on the screening strategy offered, underscoring the need for targeted interventions.

Overall, this scoping review showcases the breadth and depth of research focused on enhancing participation in cancer screening. The findings offer insights into the effectiveness of various interventions across different populations and settings, emphasizing the importance of tailored strategies to address specific barriers to screening adherence. This comprehensive examination of the literature underscores the need for ongoing research and innovation to improve cancer screening participation rates and ultimately enhance public health outcomes.

This review offers valuable insights but has limitations. It relies heavily on studies from Europe and Canada, which may not fully reflect Little Haiti’s unique context. Additionally, specific data on this community are scarce, with much of the existing information drawn from broader regional studies. The review also overlooks individual factors such as personal health beliefs, stigma, and mistrust of the healthcare system, which are critical to understanding screening behavior. Lastly, by emphasizing systematic reviews and meta-analyses, it may have missed qualitative studies that provide deeper insight into community-specific challenges.

Gaps in research and future research directions

Although studies exist that examine a variety of interventions to improve cancer adherence rates, there has been little research on specific resource-limited communities similar to Little Haiti. Little Haiti represents a subsection of the Miami-Dade community that is diverse and boasts incredible cultural richness. Few studies have explored the existing cancer screening disparities or interventions currently implemented to narrow those disparities. Thus, this scoping review hopes to highlight the gaps in research that require further investigation, such as the effect of offering a variety of screening modalities on screening adherence in Little Haiti. These modalities may include utilizing interviewing frameworks as evaluated positively by the AGREE II framework, implementing at-home testing kits, and utilizing community-specific resources such as Florida International University’s (FIU) Green Family Foundation Neighborhood Health Education Learning Program (NHELP) to improve cancer adhering screening rates.

Future research in Little Haiti should focus on identifying and addressing socio-economic and cultural barriers to cancer screening adherence. Priorities include developing and evaluating culturally tailored interventions that consider language, cultural beliefs, and health literacy. Studies should assess the impact of mobile health units and community-based participatory approaches, such as FIU’s NHELP program, in improving screening access. Additionally, research should explore technology-driven strategies, including computer-tailored interventions and motivational interviewing, to enhance adherence.

Investigations should examine the effectiveness of native-language educational programs, particularly in Haitian Creole, in dispelling misconceptions about cancer screening. Longitudinal studies are crucial to assessing the sustainability and long-term impact of these interventions on cancer mortality. Future efforts should aim to create comprehensive frameworks integrating multiple strategies to address barriers, ensuring scalability to similar underserved communities.

## Conclusions

The analysis of cancer screening adherence in Little Haiti reveals significant socio-economic and cultural barriers contributing to lower adherence rates than national and regional averages. Little Haiti, a predominantly Black and Hispanic community, faces challenges such as higher poverty rates, lower educational attainment, and a significant proportion of uninsured residents. These factors exacerbate difficulties in accessing healthcare services, including cancer screenings. Additionally, cultural misconceptions about cancer and screenings, particularly among Haitian immigrants, contribute to skepticism and reluctance toward preventive care.

This review identified multiple factors influencing low adherence, including the invasiveness and time commitment of screenings, pain concerns (especially with mammograms), uncertainty about the necessity of screening, and both logistical and emotional burdens of potential diagnoses. These findings highlight the complexity of screening resistance, emphasizing the need for comprehensive solutions tailored to diverse barriers.

Fortunately, several proven interventions can enhance adherence. Strategies should focus on reducing structural barriers, improving provider-patient communication, and implementing culturally sensitive, linguistically appropriate community-based initiatives. Mobile health units, such as those used by Florida International University’s NHELP program, can improve access to screenings and follow-up care while addressing transportation and accessibility challenges.
